# Applicability of the step test for physical fitness assessment of women with chronic venous disease symptoms: a cross-sectional study

**DOI:** 10.1590/1677-5449.202200921

**Published:** 2022-12-05

**Authors:** Ícaro do Carmo Carvalho, Daniela Karina da Silva Ferreira

**Affiliations:** 1 Universidade Federal de Pernambuco - UFPE, Recife, PE, Brasil.; 2 Prefeitura da Cidade do Recife, Programa Academia da Cidade, Recife, PE, Brasil.; 3 Fundação Oswaldo Cruz - Fiocruz, Centro Pesquisas Aggeu Magalhães, Recife, PE, Brasil.

**Keywords:** varicose veins, physical fitness, physical fitness monitors, muscle strength, lower limbs

## Abstract

**Background:**

Chronic Venous Disease (CVD) can seriously impact physical fitness. Certain measures and aptitude tests can be employed to evaluate this condition in people with CVD that are simple, quick, and less expensive alternatives when compared to laboratory methods.

**Objectives:**

To evaluate the applicability of the 4-minute step test, correlating its results with those of other measures and tests used with people with CVD symptoms.

**Methods:**

Cross-sectional descriptive study carried out with 47 active women with CVD symptoms who participate in public physical exercise programs and were recruited by spontaneous demand. After clinical evaluation of disease stage, sociodemographic data were collected and calf circumference measurements, ankle goniometry, the tiptoe test, and the 4-minute step test were conducted. The women were already familiar with the apparatus used.

**Results:**

The step test showed significant correlations (p<0.05) with calf measurements (r=0.31 and 0.32), flexibility (r=0.48 and 0.47), and the tiptoe test (r=0.33 for number of repetitions and 0.42 for speed of execution), in addition to an inverse correlation with disease severity (r=-0.29). Significant correlations were also found by age group (r=0.60 and 0.54, for calf circumference in the elderly) and by classification in tests and measurements (r=0.19 for the tiptoe test, and r=0 .29, for ankle flexibility).

**Conclusions:**

The step test proved applicable and its use in conjunction with other aptitude tests offers a more complete evaluation of active women with CVD symptoms.

## INTRODUCTION

Chronic venous disease (CVD) has high prevalence and most frequently affects adult women and elderly women.^1^^,^ 2 Its signs and symptoms impact quality of life and functionality right from the initial phases and the lower limbs are particularly affected.^3^

Risk factors associated with lifestyle, such as spending long periods standing up or sitting down and in inactivity, contribute to emergence and exacerbation of CVD.^2^ This is why many different studies have investigated the effects of physical exercises in this population as a method for preventing or controlling the disease.^4^^-^ 6 These studies attempt to assess functionality of the calf muscle pump, which is musculature that is directly involved in venous return and which, once debilitated, directly contributes to emergence and exacerbation of CVD.

Measures and tests used to assess the physical fitness of these patients include calf circumference and ankle goniometry,^7^ the 6-minute walk test,^5^ the tiptoe test,^8^ and assessments that utilize complex equipment, such as plethysmography^9^ or dynamometry.^6^

The step test is widely used to conduct cardiorespiratory assessment in healthy individuals and in patients with pulmonary diseases. It is a submaximal test, but one that imposes high metabolic demand, and is used to assess physical capacity and muscle resistance, is easy to administer, is inexpensive, and offers good representativeness of activities of daily living.^10^^,^ 11 Using a step as apparatus, the test consists of performing up and down steps for a specific period of time. It can be considered an ergometric test and there are several different protocols, varying the time of execution, which may be constant or incremental.^12^

The step test could therefore be a useful option for testing people with CVD and assessing the muscular resistance of their lower limbs, considering its simple administration and low cost and people’s familiarity with the exercise.^13^^,^ 14

The objective of this study was to correlate the results of the 4-minute step test with other tests of physical fitness, with a view to its applicability for assessing women with CVD within a public physical activity program.

## METHODS

This descriptive, cross-sectional study assessed active women with self-reported signs and symptoms of CVD, such as presence of visible lower limb varicose veins, who had been participating for at least 3 months in either the Academia da Cidade Program of the city of Recife, or the Projeto Vida Ativa, run by the Universidade Federal de Pernambuco (UFPE), in the state of Pernambuco, Brazil. Recruitment was by spontaneous demand from June to August of 2019, and women were excluded if they had active ulcers (Clinical, Etiological, Anatomic, and Pathological classification [CEAP]: C6);orthopedic, neurological, or systemic diseases that affect the calf muscle pump (CMP) or cause difficulties with performing the tests; or if they had uncontrolled diabetes or hypertension.

Sociodemographic data and health variables related to risk factors for development and exacerbation of CVD (age, marital status, number of gestations, and treatments for the disease) were collected. Clinical CVD classification (CEAP) was appraised by a healthcare professional; the Brazilian version of the Venous Insufficiency Epidemiological and Economic Study - Quality of Life/Symptom (VEINES QoL/Sym) questionnaire was administered for quality of life assessment; calf circumference was measured for both legs, using an inelastic tape measure (recorded in centimeters); and goniometry of the ankle joint was conducted for both feet using a universal goniometer (recorded in degrees), to determine maximum amplitude of ankle joint movement.

**Figure 1:**
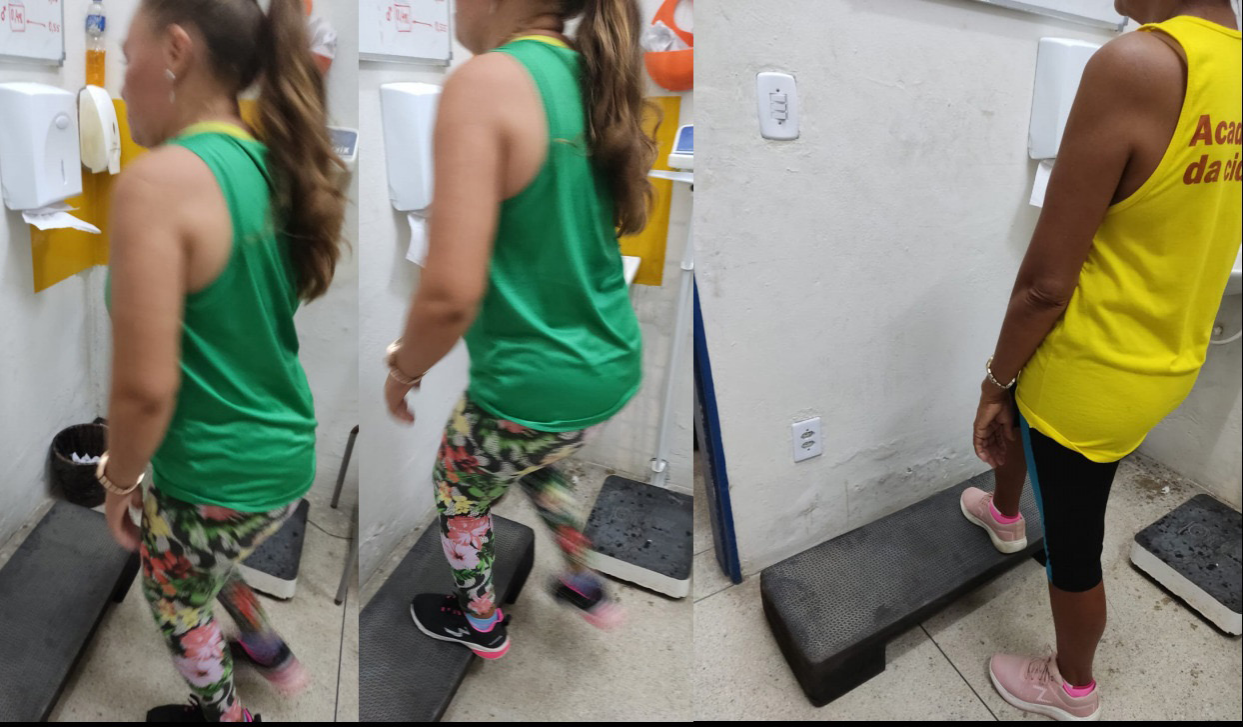
Performing the 4-minute step test.

The bilateral tiptoe test was then performed, recording the maximum number of repetitions, the time taken, and speed of execution, as described in the Monteiro et al.^15^ protocol. The step test was also conducted, using a 4-minute protocol. This begins with the subject standing in front of a 20 cm step who, after the initial command, steps up onto and down from the step as many times as possible in 4 minutes, as illustrated in Figure 1. The evaluator offers verbal encouragement while counts how many times she has stepped up onto the step. The subject is permitted to pause and restart when she feels able to do so, although the stopwatch continues running if she does so.^16^ Since the participants were already using the step as part of the activities of their programs, they were already familiar with the apparatus.

Measurements were taken and tests administered by a team of academics from the Kinesiology and Functional Assessment Laboratory at the UFPE Physiotherapy Department, under supervision by the lead researcher. Tests were administered in rotation, attempting to optimize the time taken to assess the group. All of the participants were given information about the study and the data collection procedures and signed free and informed consent forms. This study is derived from a research project approved by the Research Ethics Committee at the UFPE Health Sciences Center (decision No. 3373609).

### Data analysis

Descriptive statistics were employed for presentation of data and variables expressed as mean ± standard deviation. Normality of the distribution of the sample was demonstrated with the Shapiro-Wilk test and Pearson correlation analysis was used to relate step test results to circumference, goniometry, and tiptoe test variables. For the correlation analyses proposed in this study, adopting a medium effect size (0.3), a 0.05 alpha error, and 80% power, an ideal sample size of 84 individuals was calculated. A significance level of 0.05 was adopted. Data were analyzed using the Statistical Package for the Social Sciences (SPSS) version 25.0.

## RESULTS

**Figure 2:**
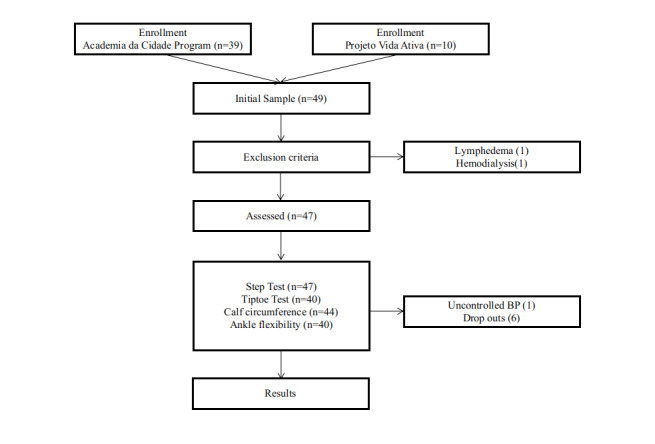
Flow diagram of study phases. BP = blood pressure.

A total of 47 women with CVD were analyzed, with a mean age of 54 years (±12 years) and with overweight/obesity (mean body mass index [BMI] of 27.4±3.9 kg/m), 37 of whom were Academia da Cidade Program users and ten of whom were participants of the Projeto Vida Ativa. Table 1 lists the general characteristics of the sample studied. During administration of the tests, one subject had an uncontrolled blood pressure event after the step test, and did not take the other tests, two subjects did not attend for goniometry or the tiptoe test, and four did not attend for the step test (Figure 2).

**Table 1:** General characteristics of the sample.

		**n**	**%**
Age group	Adults (18 to 59 years)	30	63.8
	Elderly women (60 years or over)	17	36.2
Marital status	Single	15	31.9
	Married	20	42.6
	Divorced/separated	4	8.5
	Widowed	8	17
Number of gestations	None	4	8.5
	1	9	19.1
	2	12	25.5
	3 or more	14	29.7
	Information not provided	8	17
CEAP classification	C1	8	17
	C2	16	34
	C3	10	21.3
	C4	11	23.4
	C5	2	4.3
Preferred activity	Circuit training	4	10.5
	Stretching	1	2.6
	LMR	7	18.4
	Step	20	52.6
	Running/walking	5	13.2
	Others	1	2.6
Treatment for CVD	Has been/is being treated	4	8.5
	Has not/is not being treated	43	91.5

CEAP = Clinical, Etiological, Anatomic, and Pathological classification, varying from C0 (lowest severity) to C6 (greatest severity); CVD = chronic venous disease; LMR = localized muscle resistance.

Table 2 lists the mean values for the volunteers’ calf circumference, ankle goniometry, and physical fitness test results.

**Table 2:** Results obtained for circumference and goniometry measurements and physical fitness tests.

	**N**	**Mean**	**Median**	**Standard deviation**	**Min.**	**Max.**	**Confidence interval (95%)**
Right calf (cm)	44	36.6	36.5	3.2	26.0	44.0	35.6-37.7
Left calf (cm)	44	36.9	37.0	3.2	26.5	44.0	35.9-38.1
Flexibility of right ankle (°)	40	61.3	62.0	13.1	25.0	80.0	57.2-65.9
Flexibility of left ankle (°)	40	59.3	61.0	11.0	27.0	80.0	55.8-63.3
Tiptoe test (repetitions)	40	39.0	33.0	18.0	9.0	83.0	33.0-46.0
Tiptoe test (time in seconds)	40	50.0	44.0	29.0	11.0	145.0	40.0-61.0
Tiptoe test velocity (repetitions/second)	40	0.82	0.83	0.24	0.40	1.48	0.76-0.91
4-minute step test (Number of steps)	47	101.0	104.0	22.0	38.0	146.0	94.0-110.0

Participants who stated they preferred the step activity during training sessions (n = 19) achieved a mean total of 109 steps in the step test, while those that preferred other activities (n = 28) achieved a mean total of 95 steps.

Table 3 shows correlations between step test results and the other measurements and tests conducted.

**Table 3:** Correlations between calf circumference, ankle flexibility, and tiptoe test and the 4-minute step test results.

	**Correlation**	**Significance**	**N**
Right calf (cm)	0.312*	0.042	43
Left calf (cm)	0.322^*^	0.035	43
Flexibility of right ankle (°)	0.480**	0.002	39
Flexibility of left ankle (°)	0.469^**^	0.003	39
Tiptoe test (repetitions)	0.328^*^	0.041	39
Tiptoe test (time in seconds)	0.006	0.972	39
Tiptoe test velocity (repetitions/second)	0.418^**^	0.008	39

* The correlation is significant to 0.05 (both limbs);

** The correlation is significant to 0.01 (both limbs).

**Figure 3:**
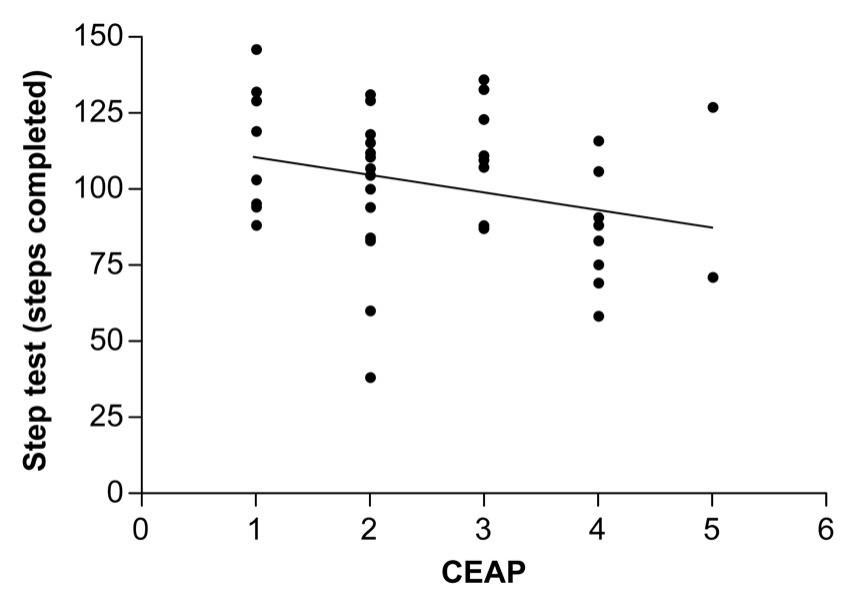
Correlation between clinical classification of chronic venous disease and performance on the step test. CEAP = Clinical, Etiological, Anatomic, and Pathological classification.

Since these tests do not have reference tables for classifying performance, the results were stratified into quartiles, revealing significant correlations (p < 0.05) with tiptoe test velocity (r = 0.19); left ankle movement amplitude (r = 0.29); and left calf circumference (r = 0.10). Analyzing the group according to their CVD clinical classifications, a significant inverse correlation (r = -0.297, p = 0.045) was detected, indicating poorer performance on the test in more severe phases of the disease, as illustrated in Figure 3.

When observed by age group, the step test had a significant correlation with the tiptoe test in adults (r = 0.48, p = 0.018) and a moderate correlation with calf circumference in elderly participants (right: r = 0.60, p = 0.014; and left: r = 0.54, p = 0.032).

## DISCUSSION

In physical fitness assessment, properties such as ease of administration and familiarity with the exercise are important to achieving trustworthy results.^17^^,^ 18 The step test offers these characteristics since it is similar to the daily living task of climbing stairs, which was confirmed in this study that observed it was easy to administer to active women who identified use of the step as their favorite activity in the physical exercise routines they perform in their exercise programs.

In individuals with CVD, the primary objective of these tests is to verify CMP functionality and its relationship with performance of activities of daily living. Instruments such as dynamometers and ultrasound are frequently employed in laboratories for this purpose when assessing this population.^6^^,^ 9 ^,^ 19 However, in addition to not being compatible with activities of daily living, their applicability to large groups may be limited by the high costs of acquisition and maintenance, in addition to the time needed for training and calibrating examiners, although equipment that can be used outside of laboratory settings does exist. Physical tests therefore constitute viable alternatives for obtaining performance data that can be used to identify the impact of CVD on individuals’ physical fitness, in addition to yielding parameters to better guide prescription of exercise training and enabling assessment of the effects of training over time.

Walking tests such as the shuttle walk test or the 6-minute walk test, are described in the literature for assessment of cardiorespiratory status and gait velocity in this population,^20^ while calf circumference measurements, ankle goniometry, and the tiptoe test are used to measure the impact of CVD on calf muscle function and ankle mobility.^21^

The step test can, therefore, be added to this list of assessments, since it employs low-cost apparatus and requires little space and a shorter time for administration, as shown by the results observed in this study, and is correlated with the results of the other tests administered to this population. The step test also proved applicable to the elderly population and demonstrated fidelity, since people with more advanced disease grades exhibit worse performance, corroborating other studies that assess the functional performance of people with CVD.^8^^,^ 22

One limitation of this study is the fact that it only assessed part of the female population already engaged in physical exercise, interfering with generalization of the results observed. The sample did not attain the ideal number of participants, and some missing data, caused by sampling losses, reduced the power of the analyses, and the sequence and order of administration of the physical fitness tests could have had an influence on the results because of the effect of tiredness. It is also important to emphasize that interpretations of the correlation coefficients can vary and the results observed in this study may be interpreted as weak or medium correlations, even those that are significant in some cases. It is thus necessary to conduct additional studies using this instrument to validate it with this population, taking into consideration variables such as age, disease severity, and physical activity level.

## CONCLUSIONS

The step test proved to be applicable for assessment of physical fitness and lower limb function in women who attend public physical exercise programs. Its use in conjunction with other tests such as the tiptoe test and goniometry offers a more complete assessment of the functional of the lower limbs, of the resistance of the calf musculature, and of the cardiorespiratory capacity of people affected by CVD, providing the necessary parameters to appropriately prescribe exercise as a coadjutant to treatment. Validation and reproducibility studies reporting reference values will enable its safe administration and replication in other scientific studies of this population.
