# Supplementary material for: Applicability of the step test for physical fitness assessment of women with chronic venous disease symptoms: a cross-sectional study
Source: J Vasc Bras. 2022 Dec 5;21:e20220092. doi: 10.1590/1677-5449.202200921 (PMC9731382; doi:10.1590/1677-5449.202200921)
Supplement: Translation

# Translation: Aplicabilidade do teste do degrau na avaliação da aptidão física de mulheres com sintomas de doença venosa crônica: estudo transversal

**DOI:** 10.1590/1677-5449.202200922

**Authors:** Ícaro do Carmo Carvalho, Daniela Karina da Silva Ferreira

**Affiliations:** 1 Universidade Federal de Pernambuco - UFPE, Recife, PE, Brasil.; 2 Prefeitura da Cidade do Recife, Programa Academia da Cidade, Recife, PE, Brasil.; 3 Fundação Oswaldo Cruz - Fiocruz, Centro Pesquisas Aggeu Magalhães, Recife, PE, Brasil.

**Keywords:** varizes, aptidão física, monitores de aptidão física, força muscular, membros inferiores

## Abstract

**Contexto:**

A doença venosa crônica (DVC) pode impactar a aptidão física dos indivíduos. Algumas medidas e testes de aptidão são aplicados para avaliar essa condição em pessoas com DVC, sendo alternativas simples, rápidas e menos dispendiosas de avaliação comparadas a métodos laboratoriais.

**Objetivos:**

Avaliar a aplicabilidade do teste do degrau de 4 minutos, correlacionando seus resultados com os de outras medidas e testes aplicados a pessoas com sintomas de DVC.

**Métodos:**

Estudo descritivo transversal realizado com 47 mulheres ativas com sintomas de DVC, participantes de programas públicos de exercícios físicos e recrutadas por demanda espontânea. Foi realizada avaliação clínica da doença, e foram coletados dados sociodemográficos, medidas de perimetria de panturrilha e goniometria de tornozelo, teste ponta do pé e teste do degrau de 4 minutos. As mulheres já eram familiarizadas com o implemento utilizado.

**Resultados:**

O teste do degrau apresentou correlações significativas (p < 0,05) com as medidas de panturrilha (r = 0,31 e 0,32), flexibilidade (r =0,48 e 0,47) e teste ponta do pé (r = 0,33 para n.º de repetições e 0,42 para velocidade de execução), além de correlação inversa com a gravidade da doença (r = -0,29). Correlações significativas também foram encontradas por faixa etária (r = 0,60 e 0,54, para perimetria de panturrilha em idosos) e por classificação nos testes e medidas (r = 0,19 para o teste ponta do pé e r = 0,29 para flexibilidade de tornozelo).

**Conclusões:**

O teste do degrau se mostra aplicável, e sua utilização, em conjunto com outros testes de aptidão, permite uma avaliação mais completa de mulheres ativas com sintomas de DVC.

## INTRODUÇÃO

A doença venosa crônica (DVC) possui alta prevalência, atingindo mais frequentemente mulheres adultas e idosas^1^^,^ 2. Seus sinais e sintomas impactam, desde suas fases iniciais, a qualidade de vida e a funcionalidade especialmente de membros inferiores^3^.

Fatores de risco associados ao estilo de vida, como longos períodos de permanência em pé ou sentado e sedentarismo, contribuem para o surgimento e agravo da DVC^2^. Por isso, vários estudos têm verificado o efeito da prática de exercícios físicos para essa população de modo a prevenir ou controlar a doença^4^^-^ 6. Nesses estudos, busca-se avaliar a funcionalidade da bomba muscular da panturrilha, musculatura diretamente envolvida no retorno venoso e que, uma vez debilitada, contribui com o aparecimento e agravo da DVC.

Entre as medidas e testes aplicados visando a avaliação da aptidão física desses pacientes, estão perimetria de panturrilha e goniometria de tornozelo^7^, teste de caminhada de 6 minutos^5^, teste ponta do pé^8^ e avaliações utilizando equipamentos complexos como pletismografia^9^ ou dinamometria^6^.

Muito utilizado para avaliação cardiorrespiratória em indivíduos saudáveis e pacientes com doenças pulmonares, o teste do degrau é um teste submáximo, mas de alta demanda metabólica, utilizado para avaliar a capacidade física e resistência muscular, sendo um teste de fácil aplicação, baixo custo e boa representatividade das atividades de vida diária^10^^,^ 11. Com uso do implemento *step*, o teste consiste na execução de movimentos de subida e descida do degrau em um determinado tempo. Podendo ser considerado um tipo de teste ergométrico, ele possui uma diversidade de protocolos, variando o tempo de execução de forma constante ou incremental^12^.

Dessa forma, pode constituir-se numa alternativa útil na avaliação de pessoas com DVC, avaliando ainda a resistência muscular de membros inferiores devido à sua fácil operacionalização, ao baixo custo e à familiaridade com o exercício^13^^,^ 14.

Assim, o objetivo deste estudo foi correlacionar os resultados do teste do degrau de 4 minutos com outros testes de aptidão física, visando sua aplicabilidade na avaliação de mulheres com DVC em um programa público de atividades físicas.

## MÉTODOS

Para este estudo, de caráter descritivo transversal, foram avaliadas mulheres com sinais e sintomas autorrelatados da DVC, como a presença visível de varizes de membros inferiores, ativas, participantes há pelo menos 3 meses do Programa Academia da Cidade do Recife (Pernambuco, Brasil) ou do Projeto Vida Ativa da Universidade Federal de Pernambuco (UFPE). O recrutamento se deu por demanda espontânea no período de junho a agosto de 2019, e foram excluídas mulheres que apresentavam úlceras ativas (classificação clínica, etiológica, anatômica e patológica [CEAP]: C6), doença ortopédica, neurológica ou sistêmica que afete a bomba muscular da panturrilha (BMP) ou dificulte execução dos testes, bem como as que estivessem com diabetes ou hipertensão descompensados. Foram coletados dados sociodemográficos e de saúde relacionados a fatores de risco para desenvolvimento e agravo da DVC (idade, estado civil, número de gestações e realização de tratamento para a doença); realizada a classificação clínica da DVC (CEAP) por profissional de saúde; aplicada a versão brasileira do questionário *Venous Insufficiency Epidemiological and Economic Study - Quality of Life/Symptom* (VEINES QoL/Sym) para avaliação da qualidade de vida; realizada a medida de perimetria da panturrilha em ambas as pernas, utilizando fita métrica inelástica (registro em centímetros); e realizada goniometria da articulação do tornozelo, com uso de goniômetro universal (registro em graus), em ambos os pés, para verificação da amplitude máxima de movimento ao nível dessa articulação. Em seguida, foi aplicado o teste ponta do pé bilateral, registrando-se o número máximo de repetições, o tempo de execução e a velocidade de execução conforme protocolo descrito por Monteiro et al.^15^; também foi feito o teste do degrau, para o qual optou-se pelo protocolo de 4 minutos. Este, com a avaliada em pé de frente a um *step* de 20 cm de altura, ao comando inicial, realizava movimentos de subir e descer o degrau o maior número de vezes possível durante 4 minutos, conforme exibido na Figura 1. O avaliador fornecia estímulo verbal, enquanto contava o número total de subidas realizadas pela avaliada. A avaliada poderia pausar a execução e retornar assim que sentir-se bem para fazê-lo, sem que o cronômetro fosse parado nessa situação^16^. Como já utilizam o *step* nas atividades dos Programas, as mulheres já estavam familiarizadas com o implemento.

**Figura 1:** Execução do teste do degrau de 4 minutos.

As medidas e testes foram aplicados pela equipe de acadêmicos do Laboratório de Cinesiologia e Avaliação Funcional do Departamento de Fisioterapia da UFPE, com a supervisão do pesquisador principal. A aplicação dos testes se deu em forma de rodízio, visando otimizar o tempo de avaliação do grupo. Todas as participantes receberam informações sobre a pesquisa e os procedimentos de coleta dos dados, assinando termo de consentimento livre e esclarecido. Este trabalho deriva de projeto de pesquisa aprovado pelo comitê de ética em pesquisa do Centro de Ciências da Saúde da UFPE (parecer n.º 3373609).

### Análise de dados

Foi utilizada estatística descritiva para apresentação dos dados e variáveis em média ± desvio-padrão. A normalidade da distribuição da amostra foi apresentada por teste de Shapiro-Wilk, e a análise de correlação de Pearson foi utilizada para confrontar os resultados do teste do degrau com as variáveis de perimetria, goniometria e teste ponta do pé. Para as análises de correlação propostas neste estudo, adotando tamanho do efeito de magnitude média (0,3), erro alfa de 0,05 e poder de 80%, foi calculada uma amostra ideal de 84 indivíduos. Foi adotado nível de significância de 0,05. Os dados foram analisados utilizando o Statistical Package for the Social Sciences (SPSS) versão 25.0.

## RESULTADOS

Foram avaliadas 47 mulheres com DVC, com idade média de 54 anos (±12 anos), com característica de sobrepeso/obesidade (índice de massa corporal [IMC] médio de 27,4±3,9 kg/m), das quais 37 eram usuárias do Programa Academia da Cidade (Recife, Pernambuco) e dez, participantes do Projeto Vida Ativa da UFPE. A Tabela 1 apresenta as características gerais da amostra estudada. Durante a aplicação dos testes, uma avaliada teve a pressão arterial descompensada após o teste do degrau, não realizando os demais, duas se ausentaram, não realizando as medidas de goniometria e o teste ponta do pé, e outras quatro se ausentaram após realizar o teste do degrau (Figura 2).

**Tabela 1:** Características gerais da amostra.

		**n**	**%**
Faixa etária	Adultas (18 a 59 anos)	30	63,8
	Idosas (60 anos ou mais)	17	36,2
Estado civil	Solteiras	15	31,9
	Casadas	20	42,6
	Divorciadas/separadas	4	8,5
	Viúvas	8	17
Número de gestações	Nenhuma	4	8,5
	1	9	19,1
	2	12	25,5
	3 ou mais	14	29,7
	Não informado	8	17
Classificação CEAP	C1	8	17
	C2	16	34
	C3	10	21,3
	C4	11	23,4
	C5	2	4,3
Atividade de preferência	Treino em circuito	4	10,5
	Alongamento	1	2,6
	RML	7	18,4
	*Step*	20	52,6
	Corrida/caminhada	5	13,2
	Outros	1	2,6
Tratamento para DVC	Fez/está fazendo	4	8,5
	Não fez/não está fazendo	43	91,5

CEAP = classificação clínica, etiológica, anatômica e patológica, variando de C0 (menor gravidade) a C6 (maior gravidade); DVC = doença venosa crônica; RML = resistência muscular localizada.

**Figura 2:** Fluxograma das etapas do estudo. PA = pressão arterial.

Nas medidas de perimetria e goniometria e nos testes de aptidão física aplicados com as voluntárias, foram encontrados os valores médios que estão apresentados na Tabela 2.

**Tabela 2:** Resultados obtidos nas medidas de perimetria e goniometria e nos testes de aptidão física.

	**N**	**Média**	**Mediana**	**Desvio-padrão**	**Mínimo**	**Máximo**	**Intervalo de confiança (95%)**
Panturrilha direita (cm)	44	36,6	36,5	3,2	26,0	44,0	35,6-37,7
Panturrilha esquerda (cm)	44	36,9	37,0	3,2	26,5	44,0	35,9-38,1
Flexibilidade do tornozelo direito (°)	40	61,3	62,0	13,1	25,0	80,0	57,2-65,9
Flexibilidade do tornozelo esquerdo (°)	40	59,3	61,0	11,0	27,0	80,0	55,8-63,3
Teste ponta do pé (repetições)	40	39,0	33,0	18,0	9,0	83,0	33,0-46,0
Teste ponta do pé (tempo em segundos)	40	50,0	44,0	29,0	11,0	145,0	40,0-61,0
Velocidade no teste ponta do pé (rep/s)	40	0,82	0,83	0,24	0,40	1,48	0,76-0,91
Teste do degrau de 4 min. (n.º subidas)	47	101,0	104,0	22,0	38,0	146,0	94,0-110,0

Entre as participantes que declararam preferência pela atividade de *step* nas sessões de treino (n = 19), a média de subidas no teste do degrau foi de 109, em contraponto às que informaram outras preferências (n = 28), cuja média foi de 95 subidas.

Buscou-se, ainda, estabelecer as correlações entre os resultados obtidos no teste do degrau com as demais medidas e testes, conforme apresentado na Tabela 3.

**Tabela 3:** Correlações entre as medidas de perimetria de panturrilha, flexibilidade de tornozelo e teste ponta do pé com o teste do degrau de 4 minutos.

	**Correlação**	**Significância**	**N**
Panturrilha direita (cm)	0,312*	0,042	43
Panturrilha esquerda (cm)	0,322^*^	0,035	43
Flexibilidade do tornozelo direito (°)	0,480**	0,002	39
Flexibilidade do tornozelo esquerdo (°)	0,469^**^	0,003	39
Teste ponta do pé (repetições)	0,328^*^	0,041	39
Teste ponta do pé (tempo em segundos)	0,006	0,972	39
Velocidade no teste ponta do pé (rep/s)	0,418^**^	0,008	39

* A correlação é significativa no nível 0,05 (duas extremidades);

** A correlação é significativa no nível 0,01 (duas extremidades).

Como os testes não apresentam tabelas de referência para classificação de desempenho, ao estratificar os resultados obtidos em quartis, também foram encontradas correlações significativas (p < 0,05) para velocidade no teste ponta do pé (r = 0,19); amplitude de movimento do tornozelo esquerdo (r = 0,29); e perimetria de panturrilha esquerda (r = 0,10). Ao analisar o grupo de acordo com a classificação clínica da DVC, foi encontrada uma correlação inversa significativa (r = -0,297, p = 0,045), indicando menor desempenho no teste quanto mais grave a fase da doença, conforme ilustrado na Figura 3.

**Figura 3:** Correlação entre a classificação clínica da doença venosa crônica e desempenho no teste do degrau. CEAP = classificação clínica, etiológica, anatômica e patológica.

Quando observadas as faixas etárias, o teste do degrau apresentou correlação significativa com o teste ponta do pé em adultos (r = 0,48, p = 0,018), bem como correlação moderada com a perimetria de panturrilha em idosos (direito: r = 0,60, p = 0,014; e esquerdo: r = 0,54, p = 0,032).

## DISCUSSÃO

Na avaliação de aptidão física, propriedades como facilidade de aplicação e familiaridade com o exercício são importantes para que se obtenham resultados fidedignos^17^^,^ 18. O teste do degrau carrega essas características ao guardar similaridade com a tarefa de vida diária de subir escadas e se ratifica neste estudo ao observar sua fácil aplicabilidade em mulheres ativas e que apontam o uso do *step* como atividade de preferência em sua rotina de exercícios físicos nos programas em que participam.

Em indivíduos com DVC, o objetivo principal desses testes é verificar a funcionalidade da BMP e sua relação com o desempenho nas atividades de vida diária. Para isso, na avaliação desse público, instrumentos como dinamômetro e ultrassom são frequentemente utilizados em laboratório^6^^,^ 9 ^,^ 19. Porém, além de não guardarem compatibilidade com as atividades práticas de vida diária, a aplicabilidade para grandes grupos pode ser prejudicada, pois demandam alto custo de aquisição e manutenção, além do tempo a ser empregado para treinamento e padronização de avaliadores, ainda que existam equipamentos que possam ser utilizados fora do ambiente laboratorial. Assim, testes físicos são alternativas viáveis para obter os dados de desempenho que permitam identificar o impacto da DVC na aptidão física do indivíduo, além de servir de parâmetro para um melhor direcionamento da prescrição do treino e para avaliar o efeito do treinamento ao longo do tempo.

Os testes de caminhada (*shuttle walk test* ou teste de 6 minutos) são encontrados na literatura para avaliação da condição cardiorrespiratória e velocidade da marcha dessa população20, e as medidas de perimetria de panturrilha, da goniometria do tornozelo e do teste de ponta de pé são utilizadas com objetivo de mensurar o impacto da DVC sobre a funcionalidade da musculatura da panturrilha e mobilidade do tornozelo^21^.

O teste do degrau pode, então, ser acrescentado a essa avaliação, uma vez que utiliza um instrumento de baixo custo, demandando pouco espaço e menor tempo de aplicação e, conforme resultados verificados neste estudo, guardando correlação com os resultados dos demais testes aplicados a essa população. Esse teste se mostra aplicável, ainda, ao público idoso, bem como se mostra fiel quando os indivíduos com graus mais avançados da doença apresentam desempenho inferior, corroborando com outros estudos que avaliam o desempenho funcional de pessoas com DVC^8^^,^ 22.

Uma limitação deste estudo é o fato de ter avaliado apenas uma parcela da população já praticante de exercícios físicos do sexo feminino, dificultando a generalização dos resultados observados. A amostra não alcançou o quantitativo ideal de participantes, e alguns dados omissos, em função de perda amostral, reduziram o poder das análises, além de que a sequência e a ordem de aplicação dos testes de aptidão física pode exercer influência nos resultados obtidos em virtude de fadiga. Ademais, é importante destacar que a interpretação dos valores de correlação pode variar e que os resultados obtidos neste estudo podem ser interpretados como correlações fracas ou médias, ainda que significativas em alguns casos. Assim, faz-se necessário que novos estudos sobre esse instrumento busquem a sua validação para essa população, levando em consideração variáveis como idade, gravidade da doença e nível de atividade física.

## CONCLUSÕES

O teste do degrau se mostra aplicável para avaliação da aptidão física e funcional de membros inferiores em mulheres usuárias de programas públicos de exercícios físicos. Sua utilização, em conjunto com outros testes como o de ponta do pé e goniometria, permite uma avaliação mais completa da condição funcional de membros inferiores, da resistência da musculatura da panturrilha e da capacidade cardiorrespiratória dessas pessoas afetadas pela doença, fornecendo os parâmetros necessário para uma prescrição adequada do exercício enquanto coadjuvante no tratamento. Estudos de validação e reprodutibilidade com apresentação de valores de referência permitirão sua aplicação segura e replicação em outros estudos científicos para essa população.
